# Longitudinal clinical phenotyping of post COVID condition in Mexican adults recovering from severe COVID-19: a prospective cohort study

**DOI:** 10.3389/fmed.2023.1236702

**Published:** 2023-08-24

**Authors:** Isaac Núñez, Joshua Gillard, Sergio Fragoso-Saavedra, Dorien Feyaerts, León Islas-Weinstein, Angel A. Gallegos-Guzmán, Uriel Valente-García, Justin Meyerowitz, J. Daniel Kelly, Han Chen, Edward Ganio, Alexander Benkendorff, Jaime Flores-Gouyonnet, Pedro Dammann-Beltrán, José Francisco Heredia-González, Gabriela A. Rangel-Gutiérrez, Catherine A. Blish, Kari C. Nadeau, Garry Nolan, Jose C. Crispín, David R. McIlwain, Brice Gaudillière, Sergio I. Valdés-Ferrer

**Affiliations:** ^1^Department of Medical Education, Instituto Nacional de Ciencias Médicas y Nutrición Salvador Zubirán, Mexico City, Mexico; ^2^Division of Postrgraduate Studies, Faculty of Medicine, Universidad Nacional Autónoma de México, Mexico City, Mexico; ^3^Section Pediatric Infectious Diseases, Laboratory of Medical Immunology, Radboud Institute for Molecular Life Sciences, Nijmegen, Netherlands; ^4^Radboud Center for Infectious Diseases, Radboud University Medical Center, Nijmegen, Netherlands; ^5^Center for Molecular and Biomolecular Informatics, Radboud University Medical Center, Nijmegen, Netherlands; ^6^Department of Anesthesiology, Perioperative and Pain Medicine, Stanford University School of Medicine, Stanford, CA, United States; ^7^Combined Study Plan in Medicine, Faculty of Medicine, Universidad Nacional Autónoma de México, Mexico City, Mexico; ^8^Department of Neurology & Psychiatry, Instituto Nacional de Ciencias Médicas y Nutrición Salvador Zubirán, Mexico City, Mexico; ^9^Department of Epidemiology and Biostatistics, UCSF, San Francisco, CA, United States; ^10^Institute for Global Health Sciences, UCSF, San Francisco, CA, United States; ^11^F.IProctor Foundation, UCSF, San Francisco, CA, United States; ^12^Department of Microbiology and Immunology, Stanford University School of Medicine, Stanford, CA, United States; ^13^Institute of Neuropathology, Faculty of Medicine, University of Freiburg, Freiburg, Germany; ^14^Department of Medicine, Stanford University School of Medicine, Stanford, CA, United States; ^15^Chan Zuckerberg Biohub, San Francisco, CA, United States; ^16^Division of Infectious Diseases, Stanford University, Stanford, CA, United States; ^17^Sean N. Parker Center for Allergy and Asthma Research, Stanford University, Stanford, CA, United States; ^18^Division of Pulmonary, Allergy, and Critical Care Medicine, Stanford University, Stanford, CA, United States; ^19^Institute for Immunity, Transplantation, and Infectious Diseases, Stanford University, Stanford, CA, United States; ^20^Department of Pathology, Stanford University School of Medicine, Stanford, CA, United States; ^21^School of Medicine and Health Sciencies, Tecnologico de Monterrey, Mexico City, Mexico; ^22^Department of Immunology and Rheumatology, Instituto Nacional de Ciencias Médicas y Nutrición Salvador Zubirán, Mexico City, Mexico; ^23^Center for Biomedical Science, Feinstein Institutes for Medical Research, New York, NY, United States

**Keywords:** post-COVID-19 conditions, long COVID, persistent COVID, SARS-CoV-2, Mexico

## Abstract

**Introduction:**

Few studies have evaluated the presence of Post COVID-19 conditions (PCC) in people from Latin America, a region that has been heavily afflicted by the COVID-19 pandemic. In this study, we describe the frequency, co-occurrence, predictors, and duration of 23 symptoms in a cohort of Mexican patients with PCC.

**Methods:**

We prospectively enrolled and followed adult patients hospitalized for severe COVID-19 at a tertiary care centre in Mexico City. The incidence of PCC symptoms was determined using questionnaires. Unsupervised clustering of PCC symptom co-occurrence and Kaplan–Meier analyses of symptom persistence were performed. The effect of baseline clinical characteristics was evaluated using Cox regression models and reported with hazard ratios (HR).

**Results:**

We found that amongst 192 patients with PCC, respiratory problems were the most prevalent and commonly co-occurred with functional activity impairment. 56% had ≥5 persistent symptoms. Symptom persistence probability at 360 days 0.78. Prior SARS-CoV-2 vaccination and infection during the Delta variant wave were associated with a shorter duration of PCC. Male sex was associated with a shorter duration of functional activity impairment and respiratory symptoms. Hypertension and diabetes were associated with a longer duration of functional impairment. Previous vaccination accelerated PCC recovery.

**Discussion:**

In our cohort, PCC symptoms were frequent (particularly respiratory and neurocognitive ones) and persistent. Importantly, prior SARS-CoV-2 vaccination resulted in a shorter duration of PCC.

## Introduction

Severe acute respiratory syndrome coronavirus 2 (SARS-CoV-2) infection that causes coronavirus disease 2019 (COVID-19) has resulted in at least 764 million infections and 6.9 million deaths worldwide (1). Over 7 million cases and around three hundred and thirty thousand fatalities have occurred in Mexico to date (1, 2). Global collaborative efforts were rapidly mobilized to prevent or reduce the impact of COVID-19, including the development of effective vaccines within 1 year after the first case was recognized. However, while vaccination programs resulted in significant reductions in disease severity and mortality, reports of new or persistent symptoms after acute SARS-CoV-2 infection emerged in a subset of patients (3–7).

Several terms describe post-COVID-19 issues, including post-acute COVID syndrome, long COVID, post-acute sequelae of SARS-CoV-2 infection (PASC), persistent COVID, or post-COVID conditions (PCC), the term we will use in this manuscript (8–11). Current reports indicate that PCC occurs between 32 and 87% of patients with COVID-19, can affect multiple organs and systems, and can last more than a year in over 25% of affected patients (12–14). Reports suggest that the incidence and duration of PCC vary according to acute COVID-19 severity, vaccination status at the time of infection, and underlying comorbidities (15, 16). Additional studies have found delayed hospitalization and female sex to be associated with PCC (17, 18).

The World Health Organization (WHO) defines PCC as an illness in people who have a history of probable or confirmed SARS-CoV-2 infection, occurring within 90 days from the onset of COVID-19, with symptoms and effects that last for at least 2 months, and where the symptoms cannot be explained by an alternative diagnosis (9). Critical questions about the duration, severity, and co-occurrence of specific PCC symptoms (9, 10). In addition, studies of PCC have largely focused on the US, European, and Chinese populations, while prospective studies in patients from Latin America are lacking (8, 19).

Early during the pandemic (June 2020), we initiated the *Longitudinal Instituto de la Nutrición-Stanford COVID-19 Collaborative Study* (LINS) to establish an exploratory longitudinal cohort of Mexican patients admitted due to COVID-19. In this exploratory study, we aimed to provide detailed clinical phenotyping, including the onset, duration, and co-occurrence of persistent symptoms to better understand PCC in an under-studied population. In addition, we analysed a set of clinical covariates to determine their association with PCC.

## Methods

### Study design and participants

We performed a prospective cohort study among adults hospitalized due to COVID-19 at Instituto Nacional de Ciencias Médicas y Nutrición Salvador Zubirán, a tertiary care, COVID-19 dedicated medical centre in Mexico City. The number of beds dedicated to non-intubated COVID-19 patients oscillated between 96 and 166 at different times during the pandemic. Intensive care units (ICUs) were expanded up to 42 beds to care for intubated patients. At the beginning of the pandemic, the hospital was fully converted into a COVID-19 centre. As with other centres in Mexico and other countries, saturation of hospital beds became a problem during peak periods and at times only the sickest people were admitted (20). COVID-19 treatments were adopted as evidence emerged. For example, dexamethasone was routinely used once the RECOVERY investigator’s announcement was made in mid-2020 (21). Other treatments such as remdesivir were only available through clinical trials and were not routinely used (22–25). Patients that were at least 18 years of age and with confirmed SARS-CoV-2 either by rapid antigen test or real-time polymerase chain reaction (RT-PCR) were deemed eligible to participate in our study. Eligible individuals were invited as soon as possible after hospital admission. Those that agreed to participate and signed informed consent were included in our study. People who were incapable of giving consent were included if the responsible family member gave consent. The first and last included cases were hospitalized on 2 July 2020, and 23 January 2022, respectively. The study investigators had no role in the care of the participants.

### Study definitions

**Figure 1:**
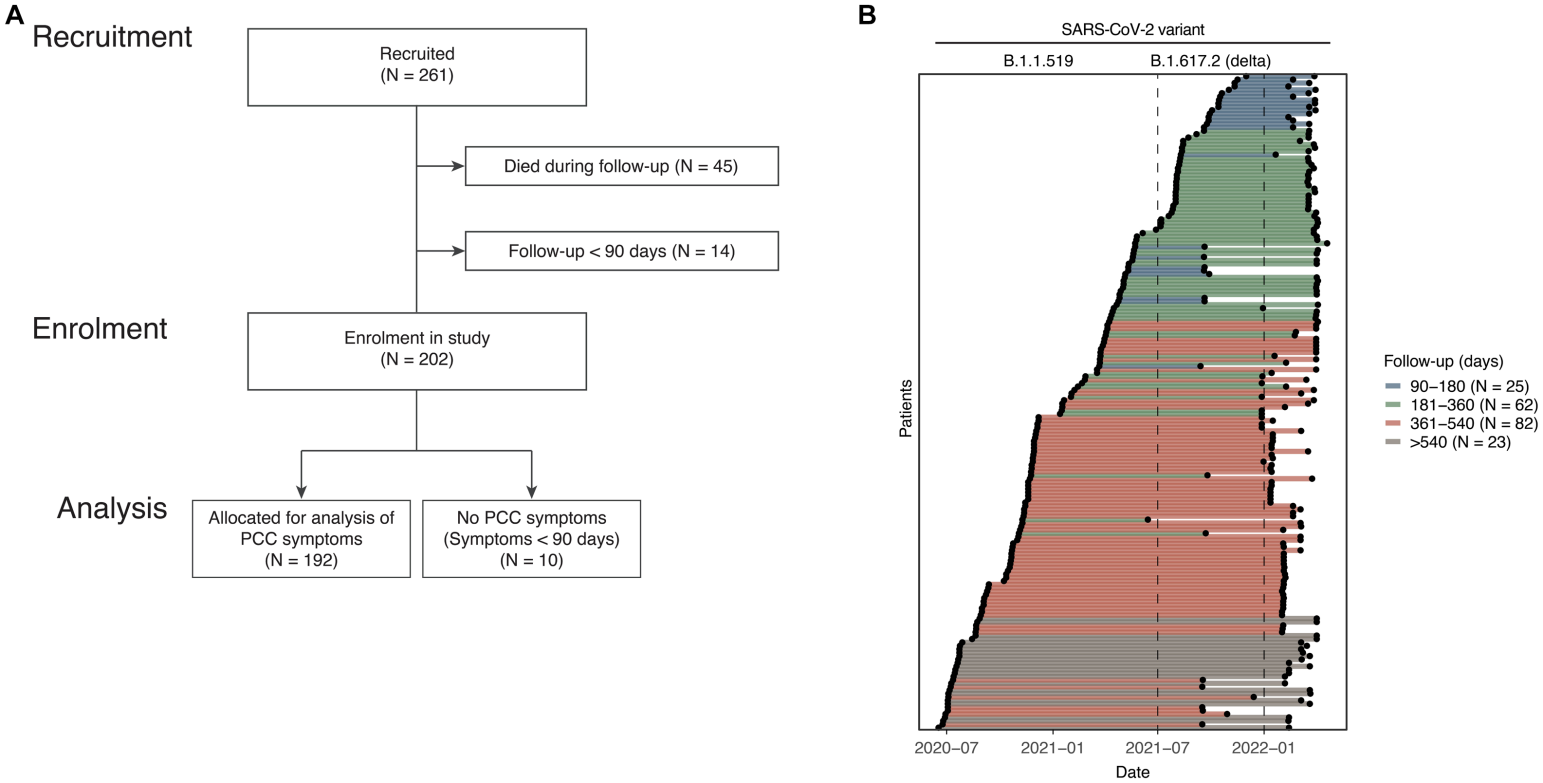
Patient selection flowchart and follow-up period. **(A)** Hospitalized COVID-19 patients were recruited in the study (*N* = 261). For analysis of PCC symptoms, deceased patients (*N* = 45) were excluded. Patients with a follow-up period of less than 90 days or patients with the longest symptom duration of less than 90 days were also excluded. **(B)** For each PCC patient (*N* = 192) (*y*-axis) the dates and length of the follow-up study period is shown (*x*-axis). The colour of each line denotes patients with follow-up periods between 90 and 180 day, between 181 and 360 days, between 361 and 540 days, or over 540 days. Indicated above are the time periods corresponding to the dominant SARS-CoV-2 variant (B.1.1.519 or B.1.617.2 (Delta)).

Using the WHO statement as a guideline, we classified patients with PCC as those patients experiencing any symptoms not present before acute COVID-19 onset, and that persisted for longer than 90 days after acute COVID-19 onset (12). Acute COVID-19 was confirmed by SARS-CoV-2 rapid antigen test or real-time polymerase chain reaction test (RT-PCR). The date of acute COVID-19 onset was retrieved from hospital charts. Upon hospital discharge, symptoms were interrogated via telephone interviews at intervals of 2 to 12 weeks, with the first call occurring 2 weeks after hospital discharge. Follow-up continued for each patient for the lengths of time indicated in Figure 1B. In cases where all PCC symptoms were reported to have ceased, no additional follow-up occurred. We report results for phone calls made up until April 4th, 2022. An 84-item questionnaire was used to collect data on clinical symptoms during phone calls. This included questions to survey the following symptoms: respiratory (use of supplemental oxygen, oxygen saturation detected by self-administered pulse oximetry: above or below 95%, nasal congestion, dyspnoea); neurological (smell disturbances, taste disturbances, persistent itch, hearing difficulties, muscle cramps); mood, sleep, and cognitive disorders (MSCD) (brain fog, insomnia, depression, anxiety, or psychosocial difficulties which were defined as a reduction in social interaction due to new-onset physical or mental limitations); gastrointestinal (diarrhoea, constipation, nausea); mucocutaneous (persistent sweating, hair loss); functional impairment (fatigue during activities of daily living, difficulties walking, using the stairs, or performing exercise). Symptoms were initially documented during the first follow-up call. Self-reported estimated dates of symptom resolution were documented during subsequent phone interviews. The duration of each symptom for each patient was defined as the time between symptom onset and symptom resolution. Alternatively, if a symptom was not resolved, the duration of each symptom was defined as the time between symptom onset and the date of the last phone call (last-alive date). We defined the total symptom duration for each patient as the longest symptom duration across all symptoms. If the total symptom duration exceeded 90 days from acute COVID-19 onset, that patient was classified as having PCC and the duration of PCC was defined as the total symptom duration. The duration of each symptom category was defined for each patient as the longest symptom duration across all symptoms within each category. Clinical and demographic variables collected were SARS-CoV-2 vaccination before hospitalization, the presence of other chronic infections, body mass index, biological sex, chronic kidney or lung disease, age, delirium during hospital stay, whether a patient was intubated or treated with dexamethasone during hospital stay, and hypertension, heart disease, or diabetes. We considered a person to be vaccinated if they received at least one dose of any SARS-CoV-2 vaccine at least 14 days before the date on which symptoms of acute infection began. Persistent low oxygen saturation after discharge was anecdotally noticed in COVID-19 survivors by study investigations before the start of this study. Thus, it was included as a possible manifestation of PCC. The predominant locally circulating SARS-CoV-2 variant for different date ranges was established based on reports from the Mexican Genomic Surveillance Consortium (COVIDGen-Mex) as follows: from study onset to July 1st, 2021, B.1.1.519 predominated; from July 2nd, 2021 to December 31st, 2022 B.1.617.2 (Delta) predominated; from January 1st, 2022 onwards B.1.1.529 (omicron) predominated (2, 26). The approximate date of exposure was estimated by subtracting 5 days from the date of symptom onset (27).

### Statistical analysis

We used descriptive statistics (count, frequency, or median with interquartile range) to summarize demographic, clinical, and hospitalization patient characteristics. To determine highly co-occurring symptoms at hospital admission or after 90 days, the number of patients with each pair of symptoms was counted and an unsupervised analysis through hierarchical clustering was performed. Symptom duration (PCC, a symptom category, or an individual symptom in a category) was analysed with the Kaplan–Meier (KM) estimator to account for follow-up periods of different lengths for patients in the cohort. We examined the effect of 16 clinical, demographic or pharmacological covariates, including age (stratified into three age groups), sex, obesity, hypertension, diabetes, delirium during the hospital stay, chronic lung, heart or kidney disease, chronic infection, intubation requirement for acute COVID-19, dexamethasone or other acute COVID-19 treatment, SARS-CoV-2 Delta variant wave, and prior SARS-CoV-2 vaccination. For each of these 16 covariates, we constructed univariable and multivariable Cox proportional hazards models and reported hazard ratios (HR) with 95% confidence intervals (higher HR = more likely to resolve PCC symptoms). Significant associations of a covariate with symptom duration were extracted (nominal *p*. value <0.05) and KM and log-rank analyses comparing patient subgroups were performed. In addition, we evaluated the proportional hazard assumption of Cox regression with log–log plots and a test of the correlation between Schoenfeld residuals and time (28). To limit potential bias due to low numbers of patients for different symptoms and covariates, we report hazard ratios of comparisons for symptoms with greater than 20 events per covariate (29). All analyses were performed with R version 3.6.1 using the ‘survival’, ‘tidyverse’, and ‘ggplot2‘ packages (30–32).

## Results

**Figure 2:**
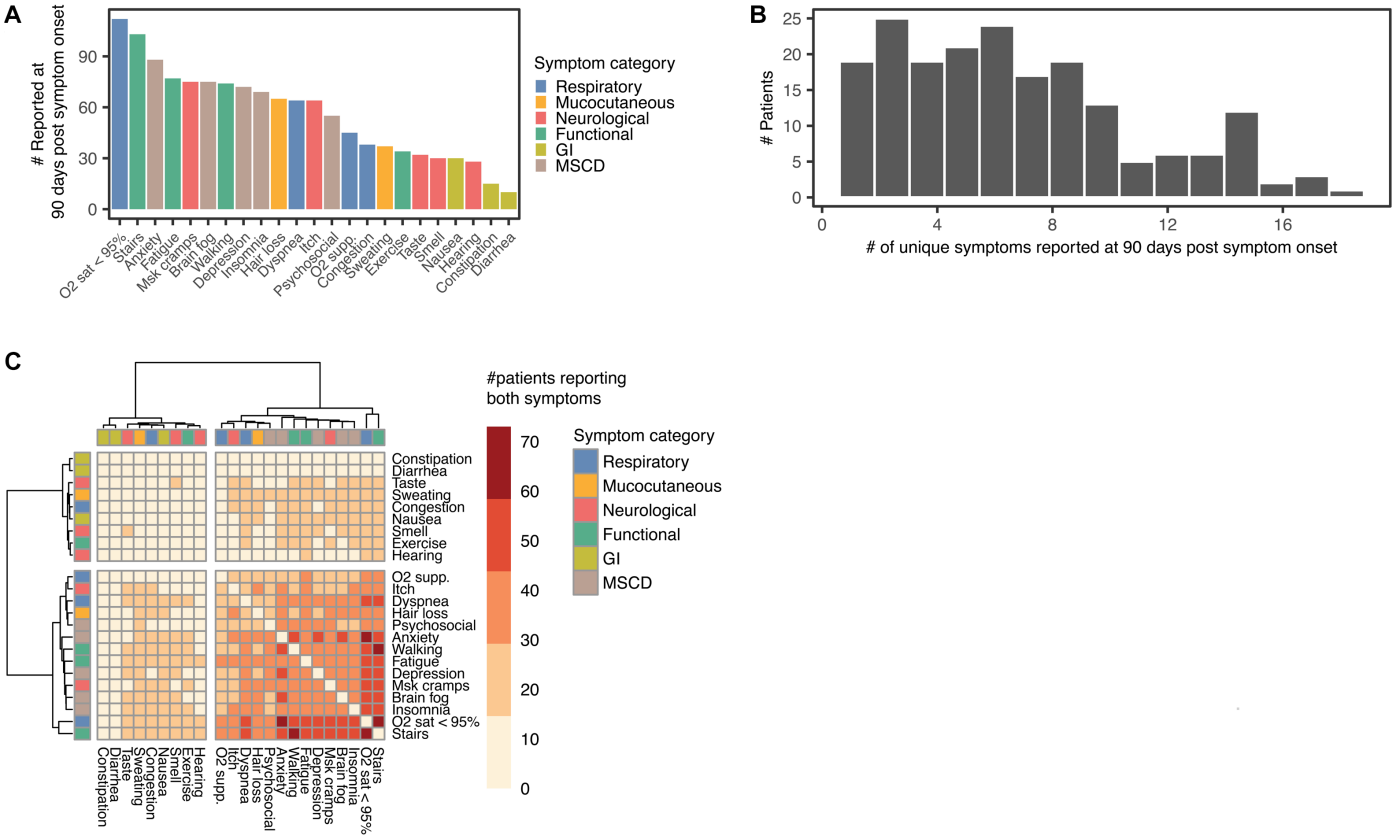
Symptoms reported by PCC patients at 90 days post hospitalization. **(A)** Number of patients reporting each symptom at 90 days post symptom onset, symptoms are coloured according to symptom category: respiratory, including O2 sat < 95%, O2 supp., dyspnoea, and congestion; Mucocutaneous, including hair loss and sweating; mood, sleep, and cognitive disorders (MSCD), including insomnia, anxiety, brain fog, depression, and psychosocial difficulties (Psychosocial); gastrointestinal (GI), including constipation, diarrhoea, and nausea; Neurological, including smell and taste disturbances (Smell and Taste), persistent itch (Itch), hearing difficulties (Hearing), and muscle cramps (MSK cramps); functional impairment (Functional), including fatigue, difficulty using the stairs (Stairs), difficulty walking (Walking), and difficulty performing exercise (Exercise). **(B)** Histogram of the number of unique symptoms reported by each patient 90 days post symptom onset. **(C)** Heatmap with hierarchical clustering of symptom co-occurrence at 90 days post symptom onset. The colour gradient of the heatmap shows the number of patients reporting both symptoms at the study start. **(A−C)** *N* = 192 PCC patients.

Two-hundred and sixty-one patients were recruited in the study between July 2nd, 2020 and January 23rd, 2022. The median time between symptom onset and recruitment into the study was 11 days (interquartile range 9–13). For the examination of PCC, we excluded 45 patients who died during the index hospitalization, 14 patients who were followed for less than 90 days, and 10 patients who did not report persistent symptoms more than 90 days after acute COVID-19. Using the WHO statement as a guideline for PCC, the remaining 192 patients were classified as PCC patients and included in subsequent PCC analysis (Figure 1A) (9). The baseline demographic and clinical characteristics of included patients are described in Table 1. Characteristics of excluded patients are described in Supplementary Table S1. Patients with PCC were recruited during the predominance of either the B.1.1.519 variant (147, 76.6%) or the B.1.617.2 (Delta) variant (45, 23.4%, Figure 1B) (26). Among patients with PCC, the median age was 53 years; 67 (65.6%) were male, and 12 (6.2%) required mechanical ventilation. Only 35 (18.2%) patients had received a dose of SARS-CoV-2 vaccine 14 or more days before the onset of COVID-19 symptoms. The median follow-up was 405 days, with a range of 91 to 626. A total of 167 (87%) PCC patients had more than 180 days of follow-up, 105 (54.7%) were followed for more than 360 days, and 23 (12%) patients had follow-up for more than 540 days (Figure 1B). Six categories of symptoms (respiratory, mucocutaneous, neurological, functional impairment, gastrointestinal (GI), mood, sleep, and cognitive disorders (MSCD)), in total comprising 23 symptoms, were assessed at study inclusion and each follow-up phone call for each patient. Low oxygen saturation was the most common symptom among PCC patients at 90 days (112, 58.3%) (Figure 2A) and during acute COVID-19 (Supplementary Figure S1A). Among the 192 PCC patients examined, 108 (56.2%) had more than five symptoms at 90 days post-symptom onset (Figure 2B), vs. 166 (86.4%) during acute COVID-19 (Supplementary Figure S1B). The most commonly co-reported symptoms at 90 days post-symptom onset were low oxygen saturation and difficulty using the stairs (*N* = 73 patients), difficulties walking and using the stairs (*N* = 64), and anxiety and low oxygen saturation (*N* = 59). Hierarchical clustering of symptom co-occurrence revealed that MSCD, functional impairment, and respiratory symptoms tended to occur together. GI and some neurological symptoms (smell, hearing, and taste disturbances) generally did not co-occur with other symptoms (at most 24 patients reporting these symptoms also reported any other co-occurring symptom, Figure 2C). Similar analyses of symptom co-occurrence during acute COVID-19 showed that respiratory and functional impairment symptoms were most reported together (Supplementary Figure S1C).

**Table 1:** Demographic and clinical characteristics of patients followed at least 90 days.

	All patients (*N* = 202)	PCC (*N* = 192)	No PCC (*N* = 10)
Male (%)	132 (65.3)	126 (65.6)	6 (60)
Age (Median, IQR)	53 (44–64)	53 (45–64)	48 (34.5–67.8)
BMI (Median, IQR)	29 (26–31.9)	29 (26–32.1)	28 (25.3–30.4)
Obesity (%)^a^	90 (44.6)	87 (45.3)	3 (30)
Prior SARS-CoV-2 vaccination (%)^b^	39 (19.3)	35 (18.2)	4 (40)
Diabetes (%)	75 (37.1)	70 (36.5)	5 (50)
Hypertension (%)	73 (36.1)	67 (34.9)	6 (60)
Heart disease (%)	1 (10.4)	19 (9.9)	2 (20)
Chronic lung disease (%)	14 (6.9)	14 (7.2)	0 (0)
Underwent invasive mechanical ventilation (%)^c^	12 (5.9)	12 (6.2)	0 (0)
Predominance of delta variant (%)^d^	49 (24.3)	45 (23.4)	4 (40)
Dexamethasone (%)	186 (92.1)	176 (91.7)	10 (100)
Chronic kidney disease (%)	21 (10.4)	18 (9.4)	3 (30)
Chronic infection (%)	6 (3)	6 (3.1)	0 (0)
Delirium (%)^e^	11 (5.4)	11 (5.7)	0 (0)

Percentages may not add up to 100% due to rounding. PCC, post COVID-19 condition.

a BMI greater than or equal to 30 Kg/m^2^.

b Any vaccine before hospital admission.

c Required mechanical ventilation.

d Determined based on the predominant variant at time of hospital admission.

e During hospital stay.

**Figure 3:**
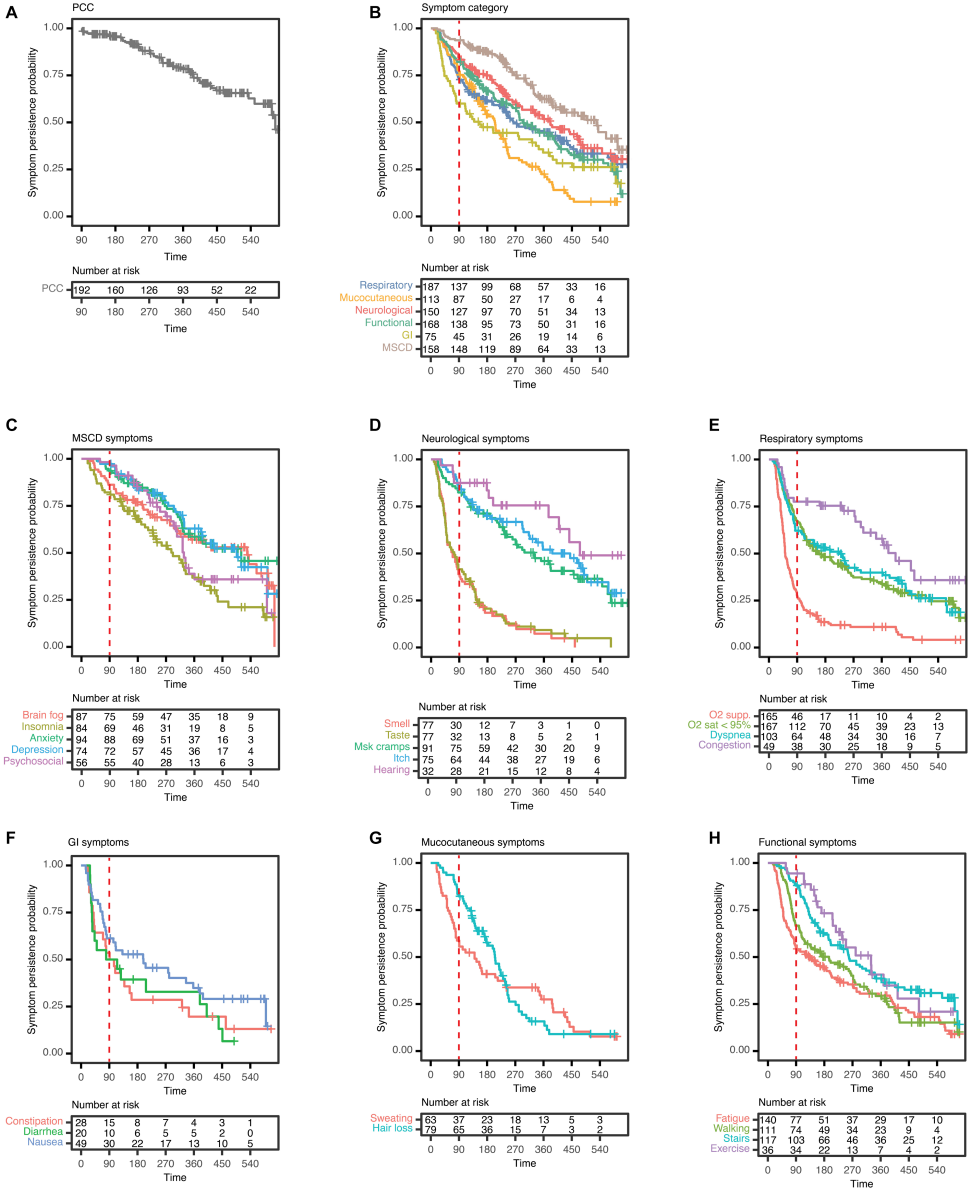
Kaplan–Meier (KM) analysis curves of symptom persistence among PCC patients. **(A)** KM analyses and risk tables of PCC; **(B)** of symptom categories; **(C)** of symptoms of mood, sleep, and cognitive disorders (MSCD), including insomnia, anxiety, brain fog, depression, and psychosocial difficulties (Psychosocial); **(D)** of neurological symptoms, including smell and taste disturbances (Smell and Taste), persistent itch (Itch), hearing difficulties (Hearing), and muscle cramps (MSK cramps); **(E)** of respiratory symptoms, including O2 sat < 95%, O2 supp., dyspnoea, and congestion; **(F)** of gastrointestinal (GI) symptoms, including constipation, diarrhoea, and nausea; **(G)** of mucocutaneous symptoms, including hair loss and sweating; **(H)** and symptoms of functional impairment (Functional), including fatigue, difficulty using the stairs (Stairs), difficulty walking (Walking), and difficulty performing exercise (Exercise). The red dashed line in each plot indicates 90 days post symptom onset. **(A−H)** *N* = 192 PCC patients.

Out of 192 PCC patients, a total of 160 (83.3%) reported at least one symptom after 180 days, 93 (48.4%) after 360 days, and 22 (11.5%) after 540 days (Figure 3A). The results of KM analyses to examine the duration of PCC, symptom categories, and individual symptoms are shown in Figure 3. Persistence probabilities were interpolated from these KM curves to determine the probability of PCC, a symptom category (Supplementary Table S2), or an individual symptom persisting at 180-, 360-, and 540 days (Supplementary Table S3). The probability of PCC persisting for up to 360 days was 0.78 (Figure 3A, Supplementary Table S2). We found that MSCD symptoms were the most persistent at 180 days post-symptom onset (Figure 3B, Supplementary Table S2), of which anxiety, psychosocial difficulties, and depression were the most persistent (Figure 3C, Supplementary Table S3). MSCD symptoms were also the most persistent by 360 days (persistence probability 0.62), followed by neurological, functional impairment, respiratory, mucocutaneous, and GI symptom categories (Figure 3B, Supplementary Table S2). Anxiety, difficulty hearing, difficulty exercising, congestion, hair loss, and nausea were the most persistent in each symptom category 180 days post-symptom onset (Figures 3C–H, Supplementary Table S3). Mucocutaneous and GI symptom categories displayed the lowest persistence probability by 540 days post-symptom onset (0.08 and 0.26 respectively, Supplementary Table S2). In only 49 PCC patients (25.5%) all symptoms had resolved by the last phone call. Among those with more than 360 days of follow-up, 29 (27.6%) had resolved all symptoms. Symptom durations for each person are shown in Supplementary Figure S2.

**Figure 4:**
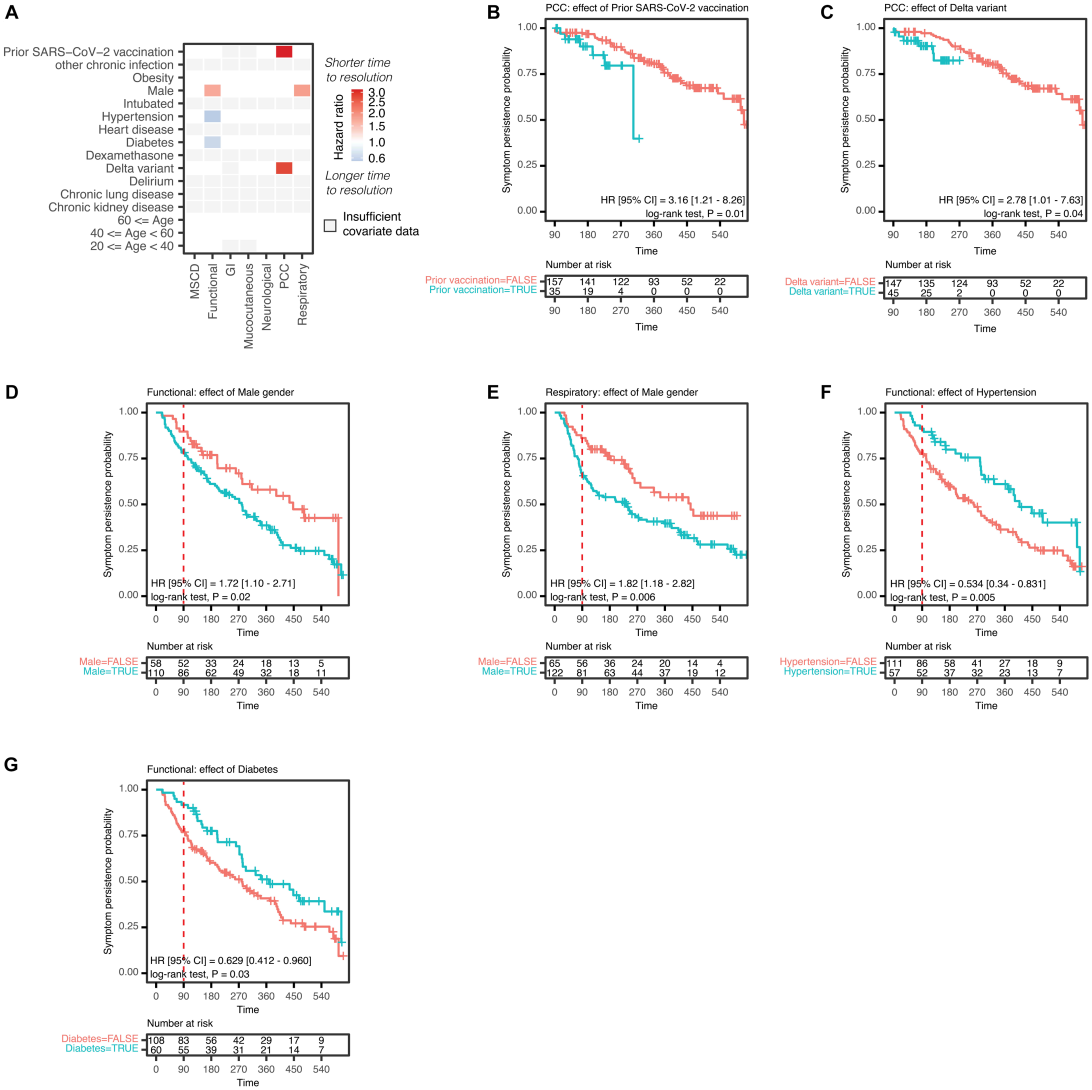
Covariate analysis of symptoms among PCC patients. **(A)** Durations of symptom categories on the *x*-axis, including mood, sleep, and cognitive disorders (MSCD), neurological, respiratory, gastrointestinal (GI), mucocutaneous symptoms, symptoms of functional impairment (Functional), and post-COVID condition (PCC) were tested for associations with patient characteristics on the *y*-axis. Patient characteristics were binary encoded as presence or absence of that characteristic for each patient and tested with a univariable Cox regression model. Associations with symptom duration are represented as the hazard ratio (HR) for the comparison of presence vs. absence of a covariate, where a HR < 1 indicates longer time to symptom resolution of the patient subgroup where a given characteristic is present. Colour gradient of HR are represented on the log2 scale. The HR of all significant associations are shown (nominal *p* value <0.05). Comparisons with fewer than or equal to 20 patients in a subgroup are indicated as ‘insufficient covariate data’. KM and log-rank analyses are shown for the following patient subgroups and symptoms: **(B)** association of prior SARS-CoV-2 vaccination and PCC; **(C)** association of Delta variant and PCC; **(D)** association of male sex and functional impairment or **(E)** respiratory symptoms; **(F)** association of pre-existing hypertension or **(G)** diabetes and functional impairment. The red dashed line in each plot indicates 90 days post symptom onset. **(A−G)** *N* = 192 PCC patients.

The effects of baseline demographic and clinical characteristics (Table 1) on symptom duration were calculated with Cox regression models and reported with HRs (14, 28). Results for PCC and symptom categories were summarized using a heatmap (Figure 4A) that depicts the HR of the group of patients presenting each covariate, where a hazard ratio less than 1 indicates a longer time to resolution if that covariate is present. Significant associations (value of *p* <0.05) with symptom duration were extracted and subgroup KM with log-rank analyses were performed (Figures 4B–G). Among PCC patients, prior SARS-CoV-2 vaccination and acute COVID-19 symptom onset occurring during a period of Delta variant predominance were associated with a shorter time to PCC resolution (Figures 4B,C). Male sex was associated with a shorter time to resolution of functional impairment and respiratory symptoms (Figures 4D,E), and a clinical history of either hypertension or diabetes (a known risk factor for PCC), was associated with a longer time to resolution of functional impairment (Figures 4F,G) (7, 15). Similarly, multivariable analysis of associations of clinical covariates with symptom duration revealed that male sex was associated with a shorter duration of functional impairment and respiratory symptoms, and hypertension was associated with a longer duration of functional impairment (Supplementary Figure S3). The number of patients with each demographic and clinical covariate is shown for each symptom category (Supplementary Figure S4). Evaluation of the proportional hazard assumption of the Cox regressions is shown in Supplementary Figure S5 (28). HRs for the resolution of PCC or of each symptom category concerning a given covariate are shown in Supplementary Figure S6. Results for the effect of covariates on individual symptoms are shown as a heatmap (Supplementary Figure S7).

## Discussion

PCC is emerging as a considerable cause that may disproportionately burden the healthcare system of Latin American countries (9, 11). We performed detailed and longitudinal phenotyping of multiple PCC symptom dimensions in a cohort of Mexican patients with PCC followed clinically for a period of up to 2 years after hospitalization for COVID-19. Several major themes emerged from the analysis of individual PCC symptom prevalence, co-occurrence, and duration. (1) While most patients with PCC reported several persistent symptoms after acute COVID-19, the duration of individual PCC symptom categories varied substantially from patient to patient, (2) respiratory, neurological, MSCD, and functional impairments contributed the most to the persistence of PCC, and (3) prior SARS-CoV-2 vaccination and infection during the Delta variant wave were associated with shorter duration of PCC symptoms.

In our cohort of patients hospitalized with COVID-19, PCC was surprisingly common, with 95% of enrolled surviving patients affected. Given we only evaluated patients hospitalized with severe COVID-19, the high incidence of PCC observed is likely to represent an overestimation of the overall incidence of PCC in people who had COVID-19. For example, a study performed in Mexico using data from the national health survey (ENSANUT) found a prevalence of 4.5% of symptoms beyond 3 months (33). However, the cross-sectional studies are limited by the potential for recall bias, which is diminished in our study given data was prospectively collected (33, 34). However, a high frequency of PCC incidence was reported in a landmark study by Carfi et al. where almost 90% of patients from an Italian post-COVID clinic had persistent symptoms 60 days after acute symptom onset (5). Among studies with long-term follow-up ≥6 months, a high proportion of persistent symptoms is uniformly reported (13, 18). The PHOSP-COVID study of discharged COVID-19 patients in the United Kingdom and an additional study by Seeßle et al. found a low proportion of recovery at 1 year (28.9 and 22.9%, respectively), while a study by Huang et al. reported 51% recovery at 1 year (13, 17, 18). These studies found fatigue as the most common symptom at 1 year, which contrasts with the high burden of low-oxygen saturation, anxiety, and difficulty using the stairs in our cohort (13, 14, 17, 18). A study derived from chart data from more than 1.2 million COVID-19 survivors showed differential recovery rates for specific PCC symptoms, where cognitive disorders, psychotic disorders, and epilepsy or seizures persisted longer than mood and anxiety disorders (35). Thus, the long-term symptoms appear to vary according to the studied population. Unlike other studies in non-Hispanic populations, we did not observe a correlation between age and an increased risk of developing PCC (7). Interestingly, a study by Jia et al. which had a high proportion of Hispanic patients (around 40%), also did not find a higher risk of PCC according to age (14). Our study, however, had relatively few people of younger age, which is concordant with the admission of sicker individuals who were usually older (36). Thus, a larger number of people of lower age may be needed to detect a correlation between age and PCC in our population.

To date, there are no targeted treatments for PCC, and interventions remain symptom-based. Our results indicate that in the cohort examined, having received prior SARS-CoV-2 vaccination may accelerate the resolution of PCC, as observed in other studies (37–39). Taken together, these results suggest vaccination may not only protect against severe acute disease but also reduce the length of PCC.

Currently, the precise causes of PCC are unknown (40). Recent studies have begun to investigate the role of genetic diversity as a possible causal factor (41). This emphasizes the importance of studies that examine PCC in different ethnic populations.

### Limitations

Our study has several limitations. Study power/sample size calculations were not performed given the explorative nature of this study and the lack of reliable data on PCC prevalence when it was designed. Thus, the relatively modest number of patients recruited at a single centre may have obscured important associations. To address this limitation, we report all significant effects of univariable analyses (nominal *p*. value <0.05) and additionally highlight those associations. We also report the results of multivariable analyses quantifying potential confounding associations between independent variables and outcomes. While less stringent, this approach highlights important associations of clinical covariates with PCC and symptom duration. For example, our result that prior SARS-CoV-2 vaccination is associated with shorter PCC duration does not cross significance in multivariable analyses. However, our result is supported by those of a recent systematic review reporting a similar pattern in other cohorts (38). Our results show that the Delta variant (considered to have enhanced virulence) was associated with a shorter duration of PCC (42–44). The coincidence with SARS-CoV-2 vaccination with the Delta variant wave is a plausible explanation for this finding, as 30 out of 45 PCC patients (66.7%) with acute infection during the Delta variant wave were previously vaccinated. Future studies will be necessary to establish the causative nature of observed interaction between the Delta variant and PCC duration as recent studies have provided conflicting results. In a nationwide study in Israel, sequelae of PCC in unvaccinated individuals were compared between Delta variant infections and those of wild-type or alpha variants, but no significant differences were reported (45). One large epidemiological study in the United Kingdom reported a significantly higher proportion of PCC patients who had acute COVID-19 during the Delta period compared to those during the Omicron period, after controlling for age and vaccination status (46). Another limitation of the study is our inability to include all COVID-19 patients that were admitted to our centre for various reasons including the lack of willingness of some patients to participate, and patients with conditions interfering with their ability to provide informed consent. Information was not available for those people hospitalized at this centre due to COVID-19 who were not enrolled in our study (screening failures); thus, we cannot determine the extent to which selection bias influenced our results. Also, the proportion of people that developed PCC could be slightly overestimated if we assumed that the 10 persons with insufficient follow-up may have not developed PCC. However, this would only represent a very slight overestimation and would not change the conclusions of our study. All new symptoms occurring after SARS-CoV-2 infection were assumed to have resulted from SARS-CoV-2 infection, possible alternative diagnoses were not evaluated. This sample selection process and not ascertaining alternative diagnoses could overestimate the proportion of patients with PCC in our study. Mexico City is located at an altitude of 2,240 m above sea level, which may result in slightly lower oxygen saturation levels than for individuals closer to sea level, which may lead to an overestimation of low oxygen saturation in our cohort (47).

In conclusion, the occurrence of PCC was almost universal and only one out of four patients had resolved symptoms by the time of last medical contact. However, there was substantial patient-to-patient variability in categories and durations of symptoms. While not immediately generalizable to the entire Mexican population, these results highlight the potential high burden of PCC and the likely importance of PCC-related clinical care in Mexico. Additional studies are required to understand the mechanistic basis of PCC, determine the long-term impact of incapacitating symptoms, develop predictive models for use in clinical practice, and create therapies to prevent, mitigate or resolve PCC.

## Data availability statement

The raw data supporting the conclusions of this article will be made available by the authors, without undue reservation.

## Ethics statement

The studies involving humans were approved by Comité de Investigación en Humanos, INCMNSZ. The studies were conducted in accordance with the local legislation and institutional requirements. The participants provided their written informed consent to participate in this study.

## Author contributions

BG and SV-F: conceptualization. IN, JG, SF-S, DF, JM, JK, HC, EG, CB, KN, GN, JC, DM, BG, and SV-F: methodology. SF-S, DF, JM, JK, HC, EG, and AB: investigation. LI-W, AG-G, UV-G, JF-G, JH-G, and GR-G: data collection. IN, JG, and LI-W: data curation. IN and JG: formal analysis and writing – original draft. LI-W, CB, KN, DM, BG, and SV-F: project administration. GN, BG, and SV-F: funding acquisition. IN, JG, SF-S, DF, LI-W, AG-G, UV-G, JM, JK, HC, EG, AB, JF-G, PD-B, JH-G, GR-G, CB, KN, GN, JC, DM, BG, and SV-F: writing – review and editing. IN, JG, and LI-W directly accessed and verified the underlying data reported in the manuscript. All authors contributed to the article and approved the submitted version.

## Funding

This work was supported by the Bill and Melinda Gates Foundation COVID-19 (Pilot Award OPP1113682 to GN, DM, HC, and INV-002704 to GN); Consejo Nacional de Ciencia y Tecnología, Gobierno de México (grant A1-S-18342 to SV-F); Fundación Gonzalo Río Arronte (Grant SL00206 to SV-F); The Stanford Centre for Human Systems Immunology (to BG); CEND COVID Catalyst Award (to BG); the Doris Duke Charitable Foundation (to BG); German Academic Exchange Service with funds from the German Federal Ministry of Education and Research (to AB); the Centers for Disease Prevention and Control (contract number 75D30120C08009 to JK); The Sunshine Foundation (to KN), David A. Crown Foundation (to KN), and Sean N. Parker Foundation (to KN). National Institutes of Health (5U19AI100627-09 Systems Approach to Immunity and Inflammation COVID supplement to GN, DM, and HC); US Food and Drug Administration Medical Countermeasures Initiative (contracts 75F40120C00176 and HHSF223201610018C to GN, DM, and HC). This article reflects the views of the authors and should not be construed as representing the views or policies of the US FDA, CDC, NIH, or other funding sources listed here.

## Conflict of interest

The authors declare that the research was conducted in the absence of any commercial or financial relationships that could be construed as a potential conflict of interest.

## Publisher’s note

All claims expressed in this article are solely those of the authors and do not necessarily represent those of their affiliated organizations, or those of the publisher, the editors and the reviewers. Any product that may be evaluated in this article, or claim that may be made by its manufacturer, is not guaranteed or endorsed by the publisher.
