# Supplementary material for: Longitudinal clinical phenotyping of post COVID condition in Mexican adults recovering from severe COVID-19: a prospective cohort study
Source: Front Med (Lausanne). 2023 Aug 24;10:1236702. doi: 10.3389/fmed.2023.1236702 (PMC10505811; doi:10.3389/fmed.2023.1236702)
Supplement: Supplementary file 1 [file Data_Sheet_1.PDF]

Supplementary Materials for

Longitudinal clinical phenotyping of Post COVID Condition in Mexican adults recovering from severe COVID-19: a prospective cohort study

Isaac Núñez, Joshua Gillard, Sergio Fragoso-Saavedra, Dorien Feyaerts, León Islas-Weinstein, Angel A. Gallegos-Guzmán, Uriel Valente-García, Justin Meyerowitz, J. Daniel Kelly, Han Chen, Ed Ganio, Alexander Benkendorff, Jaime Flores-Gouyonnet, Pedro Dammann-Beltrán, José Francisco Heredia-González, Gabriela A. Rangel-Gutiérrez, Catherine A. Blish, Kari C. Nadeau, Garry Nolan, Jose C. Crispín, David R. McIlwain, Brice Gaudillière*, Sergio I. Valdés-Ferrer*
*Corresponding authors

Contact information:

Sergio I. Valdés-Ferrer. Department of Neurology & Psychiatry, Instituto Nacional de Ciencias Médicas y Nutrición Salvador Zubirán, Mexico City, 14080, Mexico. Email: sergio.valdesf@incmnsz.mx

Brice Gaudillière. Department of Anesthesiology, Perioperative and Pain Medicine, Stanford University School of Medicine, Stanford, CA 94305, USA. Email: gbrice@stanford.edu

This document includes:

Supplementary Table 1. Demographic and clinical characteristics of patients excluded from PCC description. (pg. 2)

Supplementary Table 2. PCC and symptom category persistence probabilities at 180, 360, and 540-days post symptom onset. (pg. 3)

Supplementary Table 3. Symptom persistence probabilities at 180, 360, and 540-days post symptom onset. (pg. 4)

Supplementary Figure 1. Symptoms reported by patients at time of inclusion. (pg. 5)

Supplementary Figure 2. Symptoms reported by patients during follow-up. (pg. 6-7)

Supplementary Figure 3. Multivariable analysis of PCC symptoms. (pg. 8)

Supplementary Figure 4. Patient characteristics of PCC and symptom categories. (pg. 9)

Supplementary Figure 5. Schoenfeld residuals and log-log plots of Cox regression analyses. (pg. 10)

Supplementary Figure 6. Covariate analysis of PCC and symptom categories. (pg. 11)

Supplementary Figure 7. Covariate analysis of PCC symptoms. (pg. 12)

Supplementary Table 1. Demographic and clinical characteristics of patients that were not evaluated for Post Covid Conditions.

	Died during index hospitalization	Discharged but followed for less than 90 days	Did not meet criteria for PCC
N (%)	45 (100)	14 (100)	10 (100)
Male (%)	22 (48.9)	6 (42.9)	6 (60)
Age (Median, IQR)	64 (54-77)	64 (56-71.5)	48 (34.5 – 67.8)
BMI (Median, IQR)	26.3 (24.1-30.8)	28.4 (26.9-29.7)	28 (25.3 - 30.4)
Obesity (%)[1]	14 (31.1)	3 (21.4)	3 (30)
Prior SARS-CoV-2 vaccination (%)[2]	7 (15.6)	10 (71.4)	4 (40)
Diabetes (%)	16 (35.6)	8 (57.1)	5 (50)
Hypertension (%)	25 (55.6)	9 (64.3)	6 (60)
Heart disease (%)	9 (20)	7 (50)	2 (20)
Chronic lung disease (%)	2 (4.4)	0 (0)	0 (0)
Underwent invasive mechanical ventilation (%)[3]	14 (31.1)	1 (7.1)	0 (0)
Predominance of delta variant (%)[4]	11 (24.4)	6 (42.9)	4 (40)
Dexamethasone (%)	40 (88.9)	10 (71.4)	10 (100)
Chronic kidney disease (%)	7 (15.6)	4 (28.6)	3 (30)
Chronic infection (%)	1 (2.2)	0 (0)	0 (0)
Delirium (%)[5]	11 (24.4)	0 (0)	0 (0)

Percentages may not add up to 100% due to rounding. PCC: Post COVID-19 condition.

[1]BMI greater than or equal to 30 Kg/m^2, [2]Any vaccine before hospital admission, [3]Required mechanical ventilation, [4]Determined based on the predominant variant at time of hospital admission, [5]During hospital stay.

Supplementary Table 2. PCC and symptom category persistence probabilities at 180, 360, and 540-days post symptom onset.

	180-day persistence	360-day persistence	540-day persistence
PCC	0.96	0.78	0.63
MSCD	0.87	0.62	0.45
Neurological	0.75	0.52	0.36
Functional	0.66	0.45	0.3
Respiratory	0.62	0.45	0.33
Mucocutaneous	0.54	0.22	0.08
GI	0.47	0.34	0.26

PCC: Post COVID-19 condition; GI: Gastrointestinal; MSCD: mood, sleep, and cognitive disorders

Supplementary Table 3. Symptom persistence probabilities at 180, 360, and 540-days post symptom onset.

	180-day persistence	360-day persistence	540-day persistence
Anxiety	0.86	0.59	0.46
Psychosocial symptoms	0.85	0.39	0.36
Hearing difficulties	0.84	0.76	0.49
Depression	0.83	0.63	0.42
Brain fog	0.77	0.57	0.44
Nasal congestion	0.75	0.58	0.36
Difficulty exercising	0.73	0.41	0.21
Muscle cramps	0.71	0.46	0.37
Persistent itch	0.70	0.52	0.35
Insomnia	0.68	0.37	0.21
Difficulty using stairs	0.62	0.39	0.31
Hair loss	0.58	0.16	0.09
Dyspnoea	0.53	0.4	0.26
Nausea	0.53	0.35	0.29
Difficulty walking	0.50	0.29	0.15
O_2 sat <95%	0.48	0.34	0.25
Fatigue	0.44	0.29	0.18
Sweating	0.41	0.27	0.08
Diarrhoea	0.39	0.33	0.07
Constipation	0.29	0.2	0.13
Taste disturbances	0.21	0.09	0.05
Smell disturbances	0.18	0.07	0
Use of supplementary O_2	0.14	0.11	0.04

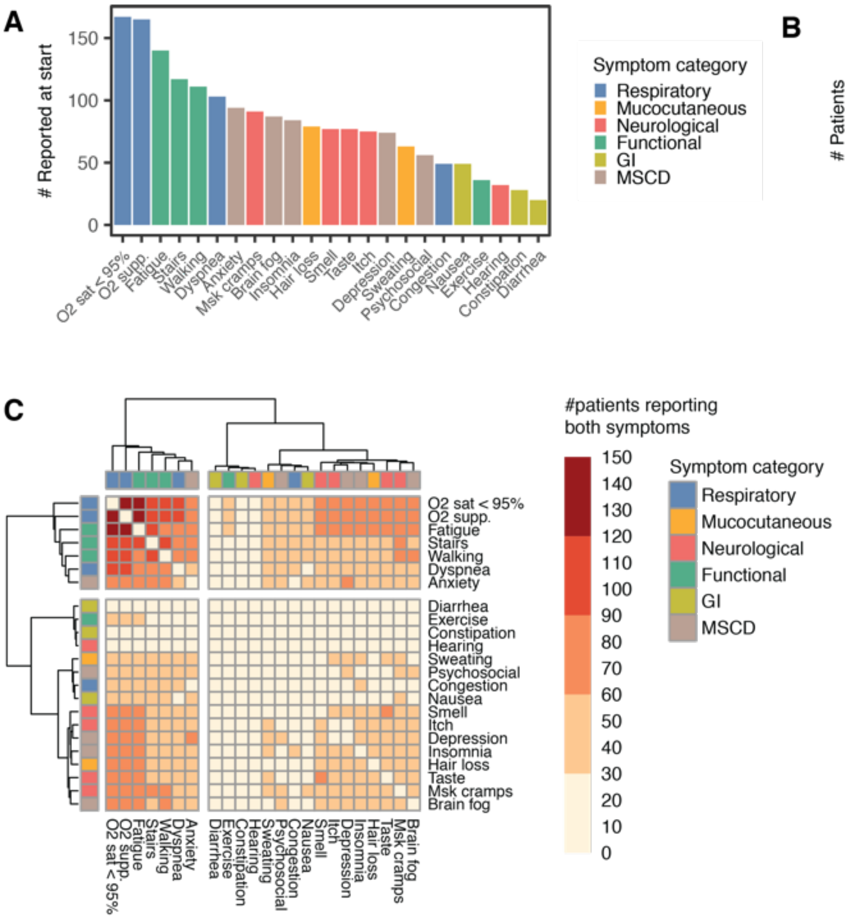

Supplementary figure 1. Symptoms reported by PCC patients at time of study inclusion. (**A**) Number of patients reporting each symptom at the time of inclusion (start), symptoms are coloured according to symptom category: Respiratory, including O_2 sat < 95%, O_2 supp., dyspnoea, and congestion; Mucocutaneous, including hair loss and sweating; mood, sleep, and cognitive disorders (MSCD), including insomnia, anxiety, brain fog, depression, and psychosocial difficulties (Psychosocial); gastrointestinal (GI), including constipation, diarrhoea, and nausea; Neurological, including smell and taste disturbances (Smell and Taste), persistent itch (Itch), hearing difficulties (Hearing), and muscle cramps (MSK cramps); functional impairment (Functional), including fatigue, difficulty using the stairs (Stairs), difficulty walking (Walking), and difficulty performing exercise (Exercise). (**B**) Histogram of the maximum number of unique symptoms reported by each patient at study inclusion. (**C**) Heatmap with hierarchical clustering of symptom co-occurrence. The colour gradient of the heatmap shows the number of patients reporting both symptoms at the study start. (A-C) N=192 PCC patients.

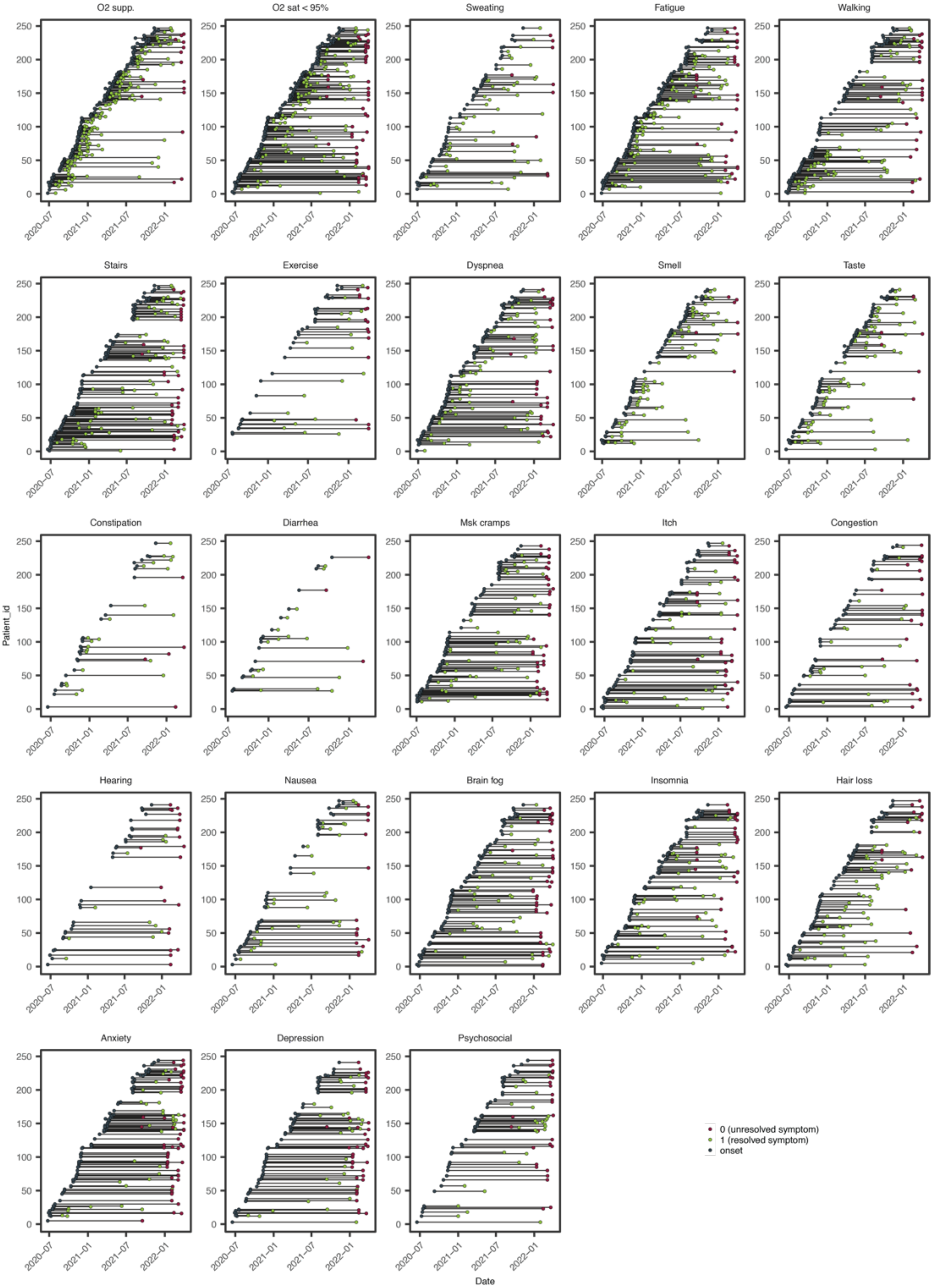
O2 supp.
O2 sat < 95%
Sweating
Fatigue
Walking
Stairs
Exercise
Dyspnea
Smell
Taste
Constipation
Diarrhea
Msk cramps
Itch
Congestion
Hearing
Nausea
Brain fog
Insomnia
Hair loss
Anxiety
Depression
Psychosocial
Patient_id
Date
0 (unresolved symptom)
1 (resolved symptom)
onset
2020–07
2021–01
2021–07
2022–01
0
50
100
150
200
250

Supplementary figure 2. Symptoms reported by PCC patients during follow-up. For each patient (y-axis) the dates and length of each reported symptom is shown. Symptom onset is indicated and symptoms are marked as unresolved at the last follow-up date or as resolved. Symptoms include: O_2 sat < 95%, O_2 supplementation, dyspnoea, congestion, hair loss, sweating, insomnia, anxiety, brain fog, depression, and psychosocial difficulties (Psychosocial), constipation, diarrhoea, nausea, smell and taste disturbances (Smell and Taste), persistent itch (Itch), hearing difficulties (Hearing), muscle cramps (MSK cramps), fatigue, difficulty using the stairs (Stairs), difficulty walking (Walking), and difficulty performing exercise (Exercise). N=192 PCC patients.

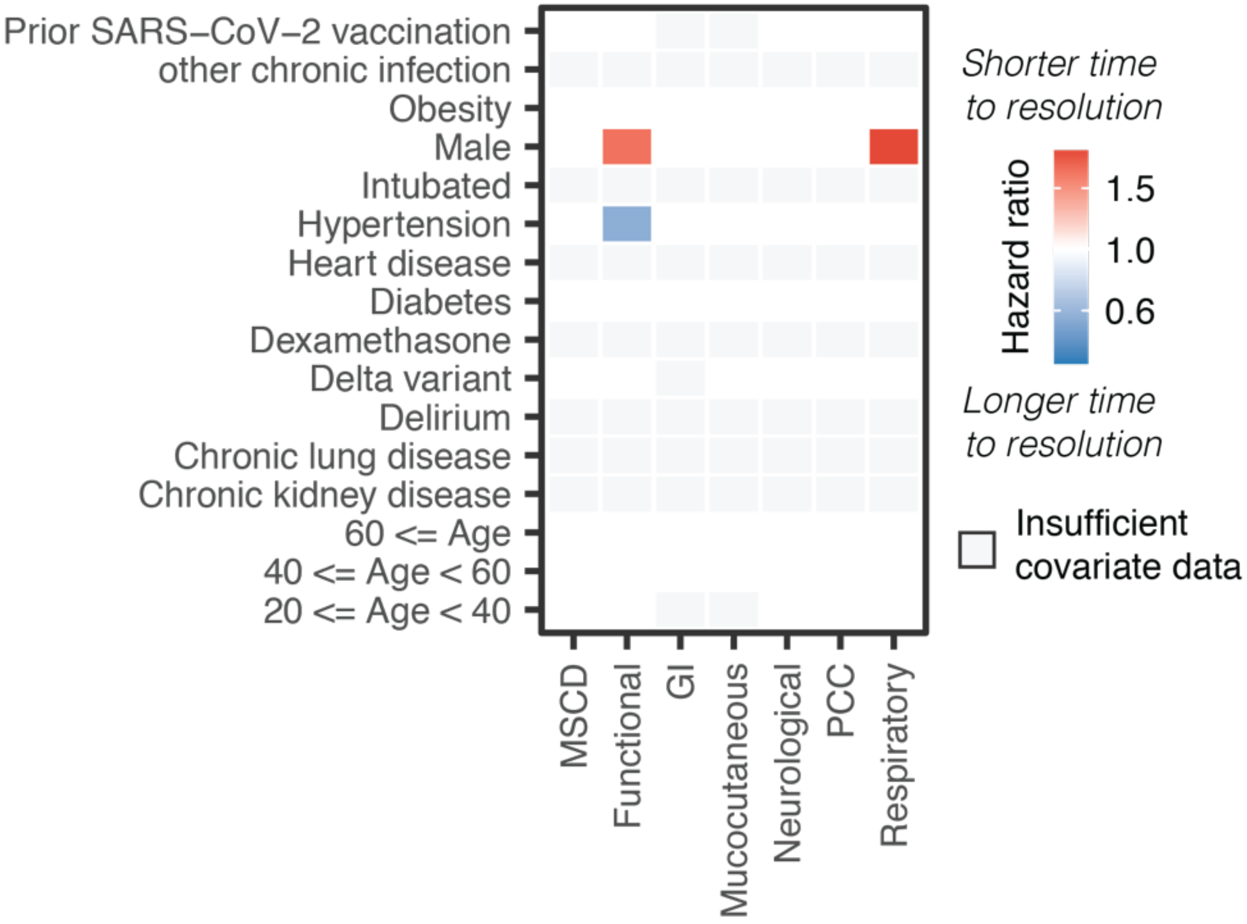

Supplementary figure 3. Multivariable analysis of symptoms among PCC patients. Durations of symptom categories on the x-axis, including mood, sleep, and cognitive disorders (MSCD), neurological, respiratory, gastrointestinal (GI), mucocutaneous symptoms, symptoms of functional impairment (Functional), and post-COVID condition (PCC) were tested for associations with patient characteristics on the y-axis. Patient characteristics were binary encoded as presence or absence of that characteristic for each patient, and tested with a multivariable Cox regression model fitting each symptom with a linear combination of all 16 patient characteristics. Effects on symptom duration are represented as the hazard ratio (HR) for the comparison of presence vs absence of a covariate, where a HR < 1 indicates longer time to symptom resolution of the patient subgroup where a given characteristic is present. Colour gradient of HR are represented on the log2 scale. The HR of all significant associations are shown (nominal *p* value < 0.05). Comparisons with fewer than or equal to 20 patients in a subgroup are indicated as 'insufficient covariate data'. N=192 PCC patients.

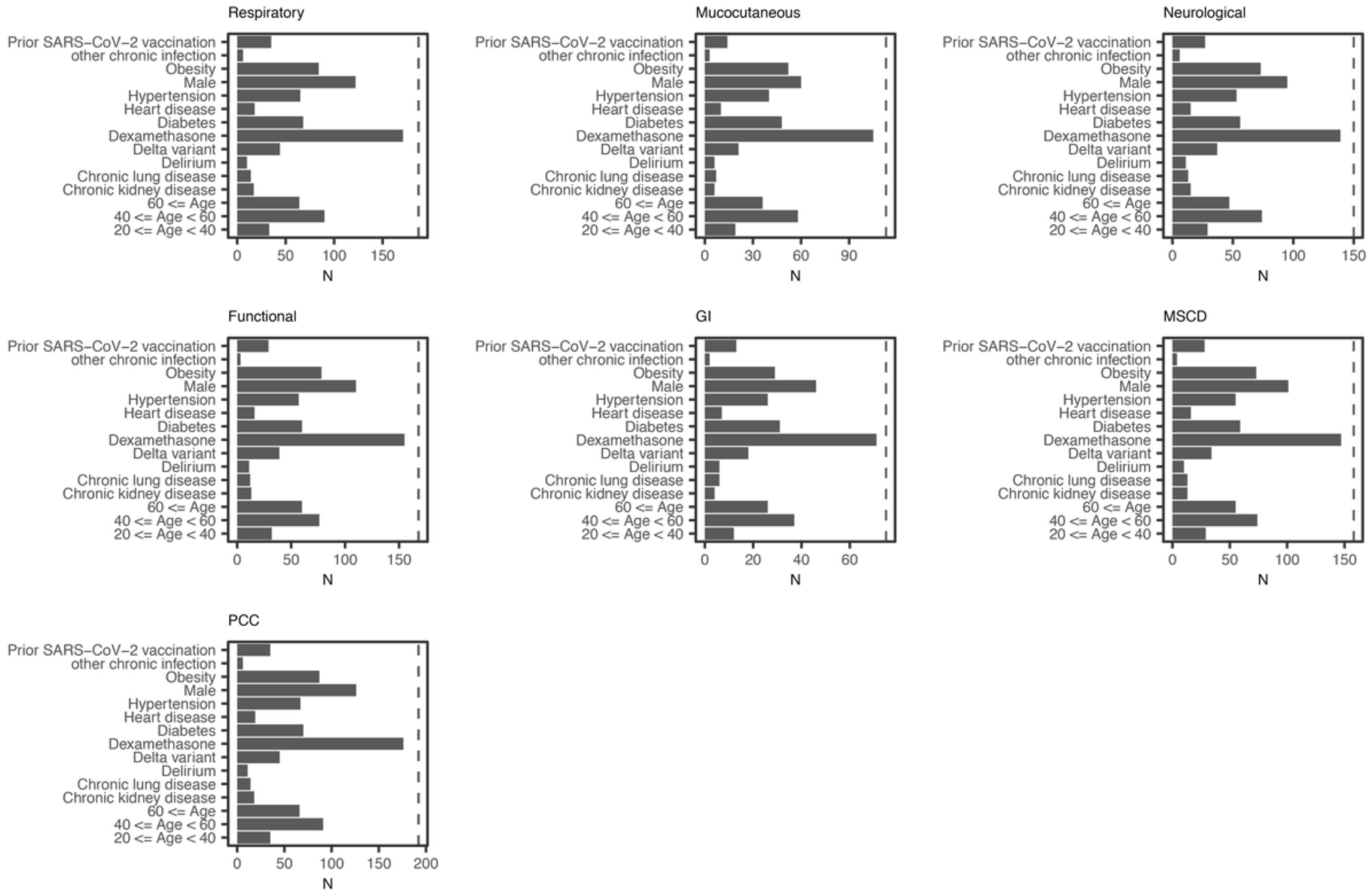

Supplementary figure 4. Characteristics of PCC patients and symptom categories. For each symptom category including mood, sleep, and cognitive disorders (MSCD), neurological, respiratory, gastrointestinal (GI), mucocutaneous symptoms, symptoms of functional impairment (Functional), and post-COVID condition (PCC), the total number of patients in each subgroup reporting that category is shown as a vertical dashed line. The number of patients displaying each characteristic (y-axis) is shown as a bar plot. N=192 PCC patients.

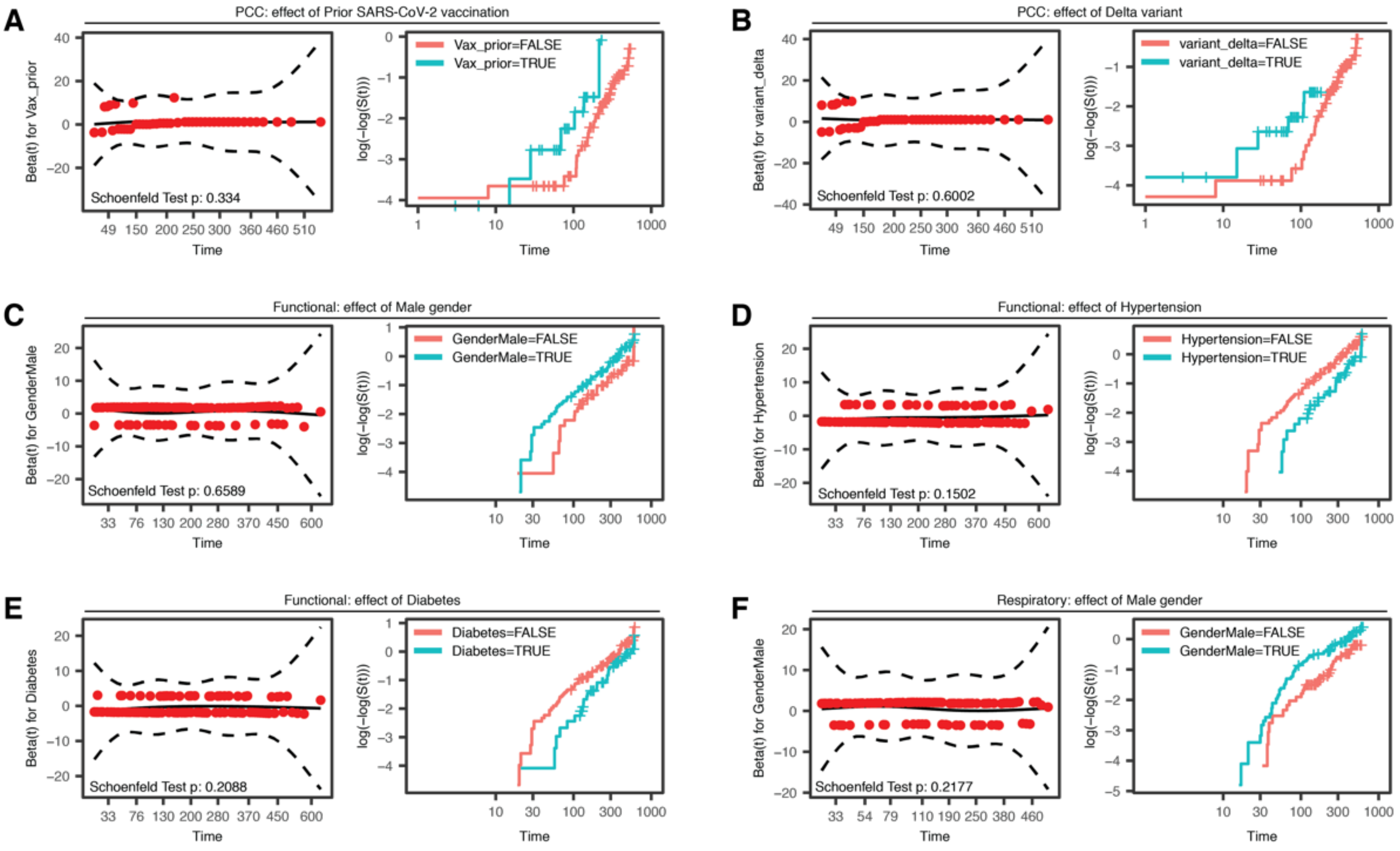

Supplementary figure 5. Schoenfeld residuals and log-log plots of Cox regression analyses. For each indicated symptom category, including mood, sleep, and cognitive disorders (MSCD), neurological, respiratory, gastrointestinal (GI), mucocutaneous symptoms, symptoms of functional impairment (Functional), and post-COVID condition (PCC), and each indicated patient subgroup, Schoenfeld residuals are shown with a test of the correlation with time (left panel) and log-log plots (right panel) are shown (related to Figure 4). (**A**) PCC: effect of Prior SARS-CoV-2 vaccination; (**B**) PCC: effect of Delta variant; (**C**) Functional: effect of Male gender; (**D**) Functional: effect of Hypertension; (**E**) Functional: effect of Diabetes; (**F**) Respiratory: effect of Male gender. (A-D) N=192 PCC patients.

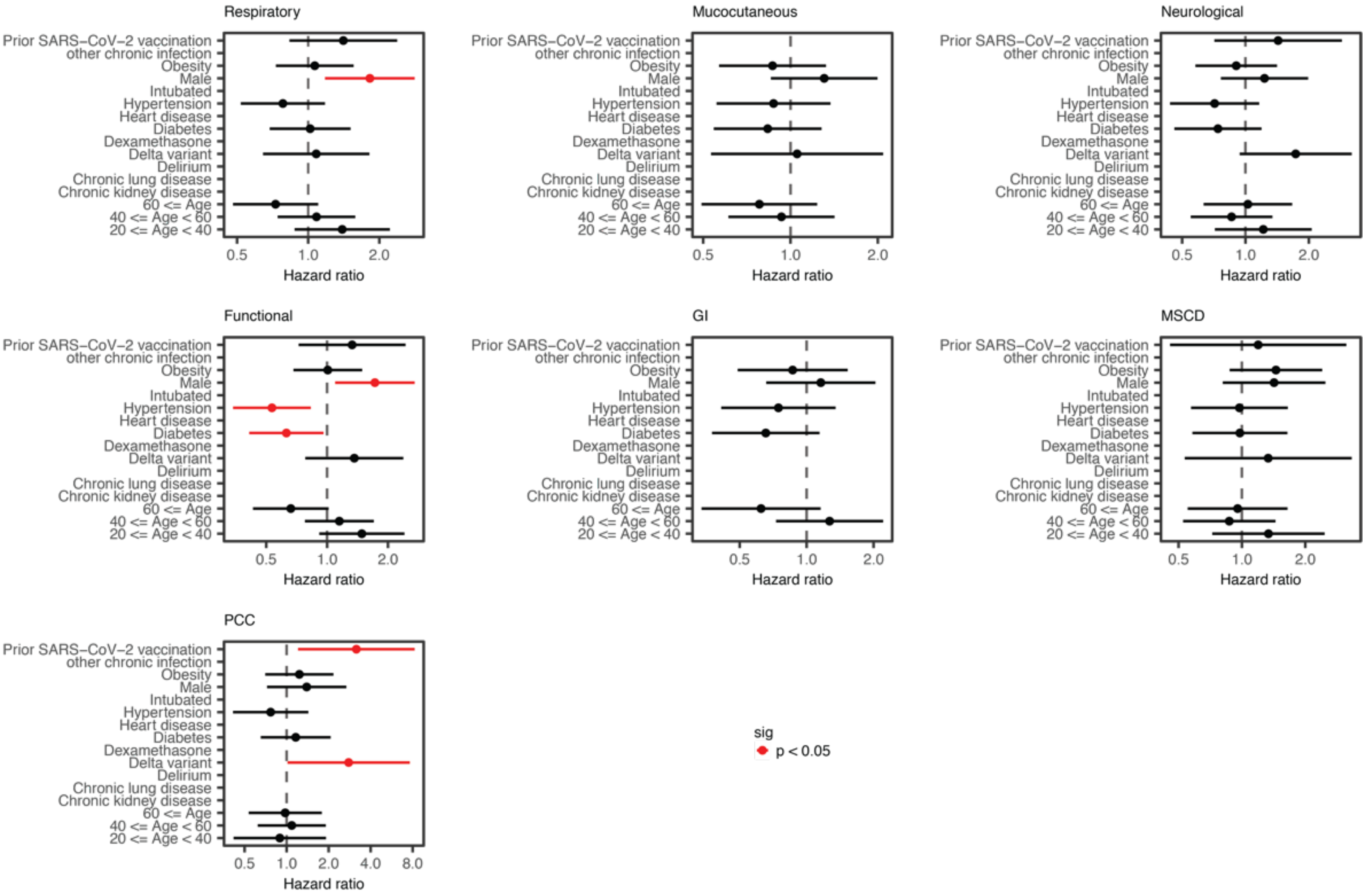

Supplementary figure 6. Covariate analysis for duration of PCC and symptom categories among PCC patients. Durations of symptom categories, including mood, sleep, and cognitive disorders (MSCD), neurological, respiratory, gastrointestinal (GI), mucocutaneous symptoms, symptoms of functional impairment (Functional), and post-COVID condition (PCC) were tested for associations with patient characteristics on the y-axis. Patient characteristics were binary encoded as presence or absence of that characteristic for each patient and tested with a univariable Cox regression model. Effects on symptom duration are represented as the hazard ratio (HR, x-axis) for the comparison of presence vs absence, where a HR > 1 indicates longer time to symptom resolution of the patient subgroup where a given characteristic is present. HR are represented on the log2 scale. The HR of all significant associations are shown (nominal *p* value < 0.05). All comparisons shown are of patient subgroups with greater than 20 patients in a subgroup. N=192 PCC patients.

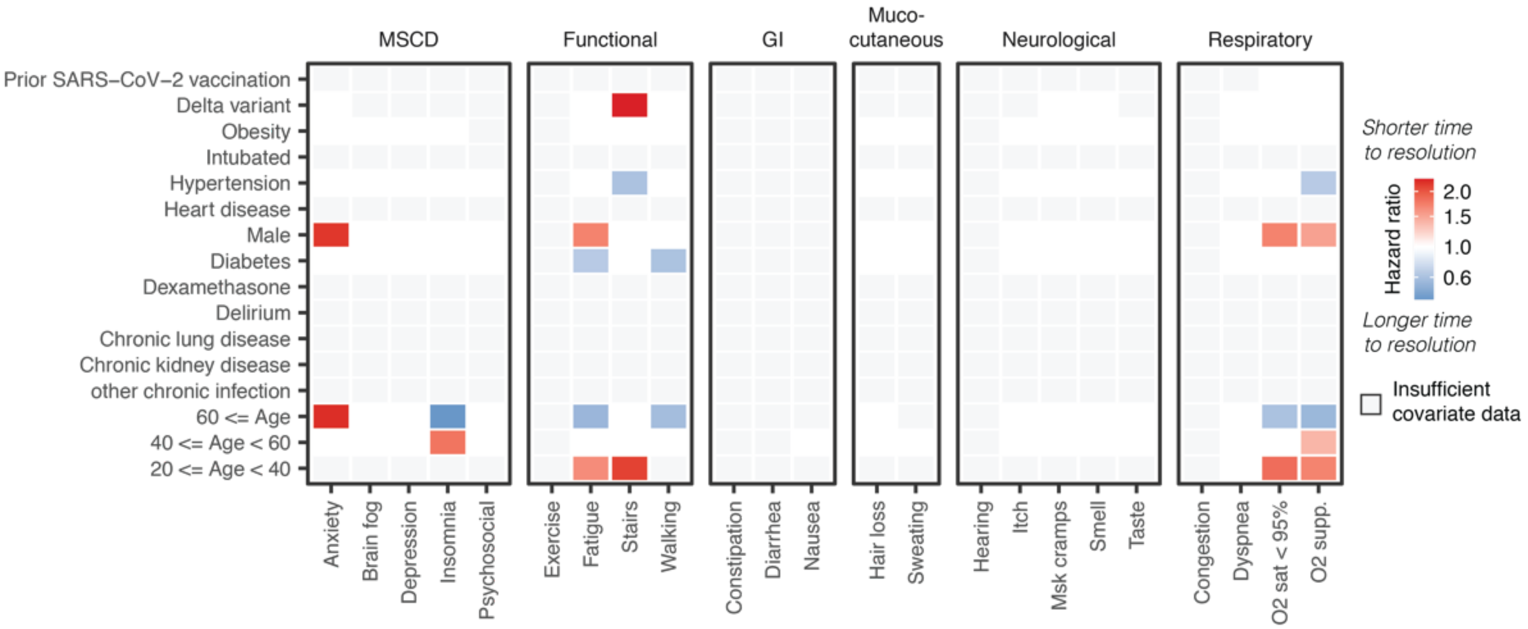

Supplementary figure 7. Covariate analysis of PCC symptoms among PCC patients. Durations of symptoms on the x-axis were tested for associations with patient characteristics on the y-axis. Symptoms are grouped into categories: Respiratory, including O_2 sat < 95%, O_2 supp., dyspnoea, and congestion; Mucocutaneous, including hair loss and sweating; mood, sleep, and cognitive disorders (MSCD), including insomnia, anxiety, brain fog, depression, and psychosocial difficulties (Psychosocial); gastrointestinal (GI), including constipation, diarrhoea, and nausea; Neurological, including smell and taste disturbances (Smell and Taste), persistent itch (Itch), hearing difficulties (Hearing), and muscle cramps (MSK cramps); functional impairment (Functional), including fatigue, difficulty using the stairs (Stairs), difficulty walking (Walking), and difficulty performing exercise (Exercise). Patient characteristics were binary encoded as presence or absence of that characteristic for each patient and tested with a univariable Cox regression model. Effects on symptom duration are represented as the hazard ratio (HR) for the comparison of presence vs absence of a covariate, where a HR < 1 indicates longer time to symptom resolution of the patient subgroup where a given characteristic is present. Colour gradient of HR are represented on the log2 scale. The HR of all significant associations are shown (nominal p value < 0.05). Comparisons with fewer than or equal to 20 patients in a subgroup are indicated as 'insufficient covariate data'. (A-C) N=192 PCC patients.
